# Fetuin-B (FETUB): a Plasma Biomarker Candidate Related to the Severity of Lung Function in COPD

**DOI:** 10.1038/srep30045

**Published:** 2016-07-22

**Authors:** Wen-qi Diao, Ning Shen, Yi-peng Du, Bei-bei Liu, Xiao-yan Sun, Ming Xu, Bei He

**Affiliations:** 1 Department of Respiratory Medicine, Peking University Third Hospital, Beijing 100191, China; 2 Department of Cardiology, Institute of Vascular Medicine, Peking University Third Hospital, Key Laboratory of Molecular Cardiovascular Sciences, Ministry of Education, Beijing 100191, China

## Abstract

Biomarkers for the progression of lung function in COPD are currently scarce. Plasma fetuin-B (FETUB) was identified by iTRAQ-based proteomics and was verified by ELISA in another group. Information regarding acute exacerbation (AE) was collected in a one-year follow-up programme. FETUB concentrations (1652 ± 427 ng/ml) were greater in COPD patients than in controls (1237 ± 77 ng/ml). The concentrations of FETUB in GOLD II (1762 ± 427 ng/ml), III (1650 ± 375 ng/ml) and IV (1800 ± 451 ng/ml) groups were greater than those in the controls (1257 ± 414 ng/ml) and the GOLD I (1345 ± 391 ng/ml) group. ROCs indicated that FETUB distinguished COPD patients from controls (AUC 0.747, 95% CI: 0.642–0.834) and also GOLD II, III and IV from GOLD I COPD patients (AUC: 0.770, 95% CI: 0.634–0.874). The combination of FETUB and fibrinogen performed better (AUC: 0.804, 95% CI: 0.705–0.881). FETUB also predicted the occurrence of AE (AUC: 0.707, 95% CI: 0.566–0.824) or frequent AE (AUC: 0.727, 95% CI: 0.587–0.840). FETUB concentrations were negatively correlated with FEV1%pred (*r* = −0.446, *p* = 0.000) and positively correlated with RV%pred (*r* = 0.317, *p* = 0.004), RV/TLC% (*r* = 0.360, *p* = 0.004), CT emphysema% (*r* = 0.322, *p* = 0.008) and grades of lung function (*r* = 0.437, *p* = 0.000). In conclusion, FETUB is likely to assist the diagnosis and management of COPD as a complement for other markers.

Chronic obstructive pulmonary disease (COPD), a complex syndrome characterized by the progressive deterioration of pulmonary function and increasing airway obstruction, causes substantial mortality and morbidity throughout the world^1^. Biomarkers are vital for the management, therapy and diagnosis of complex diseases^2^. Currently, biomarkers for the progression of lung function in COPD are scarce^3^. According to the results of the Evaluation of COPD Longitudinally to Identify Predictive Surrogate End-points (ECLIPSE), plasma fibrinogen is a relatively stable prognostic marker^4^ related to the body mass index (BMI), airflow obstruction, dyspnea, and exercise capacity (BODE) index; the exacerbation rate; and the mortality. Club cell protein-16 (CC16)^5^ showes a weak association with the severity of COPD in former smokers. Surfactant protein-D (SP-D)^6^ is weakly related to exacerbation rates of COPD. Pulmonary and activation-regulated chemokine (PARC)^7^ is weakly associated with the mortality of COPD. It is difficult to identify the progression of lung function in COPD using these biomarkers. Consequently, the identification of biomarkers for the progression of lung function is urgently needed^3^.

Biomarker identification is generally based on its potential mechanism in disease progression, and it is then are verified in a cohort. However, some important proteins may easily be ignored if their functions in the disease have not already been identified. Hence, new biomarkers should be identified by unbiased and high-throughput techniques. Proteomics has the ability to identify novel low abundant proteins and has been widely applied to disease biomarker studies especially in the tumour field^8^. Accordingly, several investigators in the respiratory field tried to find COPD biomarkers by proteomics. These studies have several proteins that distinguish COPD patients from controls^9^,^10^,^11^,^12^,^13^,^14^ as well as the clinically stable stage of COPD from acute exacerbation (AE) of COPD^15^,^16^,^17^.

Currently, proteomics-based biomarker studies in the COPD field are mainly focused on gel-based^11^,^14^, targeted^16^,^17^ and labeling-free^9^,^10^,^15^ proteomic techniques. The gel-free isobaric tags for relative and absolute quantification (iTRAQ)-based proteomic technique, an isotope-labelling and untargeted approach, have been used in the COPD field^18^. However, a previous study^18^ aimed to identify differentially expressed proteins between patients with or without COPD family history but not for clinically stable COPD patients. To our knowledge, the technique has not been used to investigate plasma biomarkers related to the clinically stable stage of COPD. Herein, we tried to identify plasma biomarkers for the progression of lung function in COPD by the gel-free iTRAQ-based proteomic technique. To further verify and investigate the clinical value of these biomarker candidates identified in the discovery group by the proteomics, we measured their levels in plasma in a larger population by enzyme-linked immuno sorbent assay (ELISA).

## Results

### Characteristics of subjects

To identify biomarker candidates, a panel of 8 male patients with COPD were recruited in the discovery group. The subjects had no diabetes mellitus, hyperlipidermia, hepatitis or hypertension. Detailed information of the subjects is shown in Table S1.

To verify the biomarker candidates, an independent panel of 87 male subjects was used for the verification group. Due to different research goals, the verification group was divided into two (control and COPD) or five subgroups (control, GOLD I, GOLD II, GOLD III and GOLD IV). Details regarding the grouping strategy and variables (age, gender, BMI, smoking history, smoking cessation, forced expiratory volume in a second (FEV1)%pred, residual capacity (RV)%pred, ratio of RV to total lung capacity (RV/TLC)%, percentage of emphysema assessed by computed tomography (CT emphysema)%, AE, frequent AE, FETUB, fibrinogen, 78 kDa glucose-regulated protein GRP-78, diabetes mellitus, hyperlipidermia, hepatitis, hypertension, abnormal alanine aminotransferase (ALT) and abnormal aspartate aminotransferase (AST)) are presented in Table 1.

According to the Shapiro-Wilk (S-W) and Levene tests, the corresponding statistical method for each continuous variable (age, gender, BMI, smoking history, smoking cessation, FEV1%pred, RV%pred, RV/TLC%, CT emphysema%, FETUB, fibrinogen or GRP-78) was used to identify the discrepancy between study groups listed in Table S2. Categorical variables (diabetes mellitus, hyperlipidermia, hepatitis, hypertension, abnormal ALT and abnormal AST) were assessed by a chi square test as shown in Table S3. Notably, age and hypertension were statistically different between COPD and controls. Moveover, age, smoking cessation and hypertension were statistically different between groups (control, GOLD I, II, III and IV). These potential confounders were corrected in the covariance of analyses and the multivariate linear regression model.

### Identification of differentially expressed proteins by the gel-free iTRAQ-based proteomic technique and verification of selected proteins by ELISA in the discovery group

The iTRAQ-based proteomic technique was conducted in the discovery group consisting of 8 subjects (n = 2, GOLD I; n = 2, GOLD II; n = 2, GOLD III; n = 2, GOLD IV). The technique identified 282,995 spectra including 23,693 unique spectra, 3369 unique peptides and 830 proteins. Appendix 1 showed 38 differentially expressed proteins identified in comparison I (GOLD II vs GOLD I). Appendix 2 showed 23 differentially expressed proteins identified in comparison II (GOLD III vs GOLD I). Appendix 3 showed 25 differentially expressed proteins identified in comparison III (GOLD IV vs GOLD I). Table 2 showed that a total of 9 proteins were simultaneously identified in appendixes 1, 2 and 3. Three proteins were increased and six proteins were decreased in the GOLD II, III and IV subgroups compared with GOLD I.

**Figure 1:**
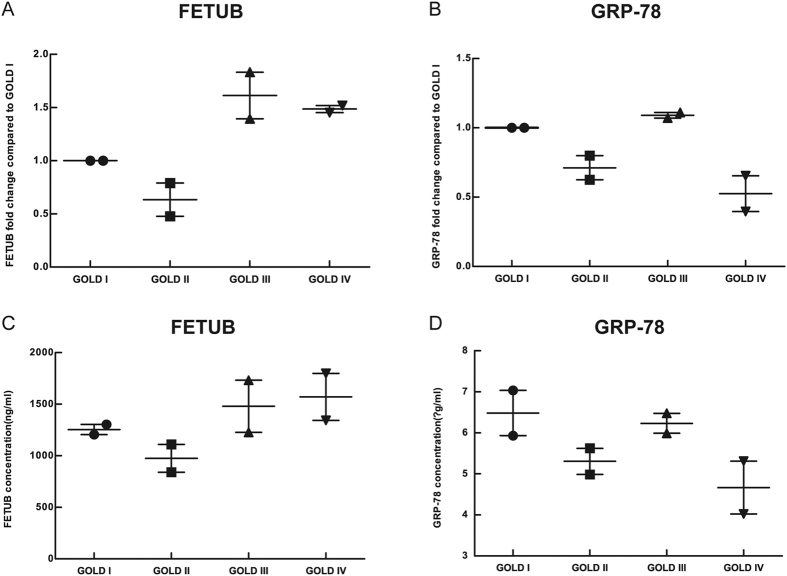
Verification of FETUB and GRP-78 by ELISA in the discovery group. Legend: (**A**) and (**B**) represented the quantified FETUB and GRP-78-fold change, respectively, compared to GOLD I by iTRAQ-based proteomics; (**C**) and (**D**) represented the concentration of FETUB and GRP-78, respectively, as determined by ELISA. Group: n = 2, GOLD I; n = 2, GOLD II; n = 2, GOLD III; n = 2, GOLD IV. The horizontal line with error bar denotes mean values (±SE). Abbreviations: GOLD, Global Initiative for Obstructive Lung Disease; iTRAQ, isobaric tags for relative and absolute quantification; ELISA, enzyme-linked immuno sorbent assay; FETUB, fetuin-B; GRP-78, 78 kDa glucose-regulated protein.

Due to the availability of kits used to measure these proteins, FETUB and GRP-78 were determined by ELISA in the discovery group. The fold changes of FETUB and GRP-78 were quantified by gel-free iTRAQ-based proteomic technique and are presented in Panel (A) and Panel (B), respectively, in Fig. 1. The concentrations of FETUB and GRP-78 measured by ELISA are presented in Panel (C) and Panel (D), respectively, in Fig. 1. The changing trends measured by ELISA were in accordance with those quantified by the iTRAQ-based proteomic technique, thereby demonstrating the reliability of the proteomic technique.

### Validation of select proteins by ELISA in the verification group

In the COPD vs Control comparison, univariate analysis indicated the mean concentration (±SD) of FETUB in COPD patients (1652 ± 427 ng/ml) was greater than that (1237 ± 77 ng/ml) in controls. Both covariance analysis and multivariate linear regression model for the COPD vs Control comparison, which corrected for age and hypertension, indicated that FETUB concentration was only affected by group (COPD vs Control).

In the GOLD I, II, III and IV vs Control comparison, univariate analysis indicated that the mean concentration (±SD) of FETUB was different statistically in all groups (controls, GOLD I, II, III and IV), and the post-hoc test indicated that FETUB concentrations of GOLD II (1762 ± 427 ng/ml), GOLD III (1650 ± 375 ng/ml) and GOLD IV (1800 ± 451 ng/ml) was significantly greater than those of the control group (1257 ± 414 ng/ml). And FETUB concentrations of the GOLD II and GOLD IV groups were significantly greater than those of the GOLD I group (1345 ± 391 ng/ml). The difference of FETUB between the GOLD I group (1345 ± 391 ng/ml) and the GOLD III group (1650 ± 375 ng/ml) were not significant statistically, which may have been due to the lack of statistical power. There were no difference significantly between the GOLD I group and the control group. Both covariance analysis and multivariate linear regression model for the GOLD I, II, III and IV vs Control comparison, which corrected for age, hypertension and smoking cessation, indicated that FETUB concentration was only affected by the group (GOLD I, II, III, IV vs Control).

The concentration of fibrinogen was different statistically between COPD patients and controls. In the GOLD I, II, III and IV vs Control comparison, only fibrinogen concentrations in the GOLD IV group were greater than those in the control group.

The concentration of GRP-78 was not significant statistically in the COPD vs controls comparison or the GOLD I, II, III and IV vs controls, comparison.

**Figure 2:**
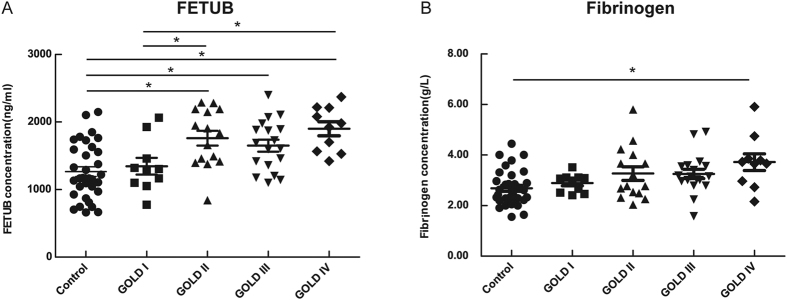
FETUB and fibrinogen concentration of COPD patients with different grades of lung function compared to controls in the verification group. Legend: n = 34 smokers without COPD; n = 53 COPD smokers. The horizontal line with error bar denotes mean values (±SE). *p < 0.05; **p < 0.01. Abbreviations: FETUB, fetuin-B; COPD, chronic obstructive pulmonary disease; GOLD, Global Initiative for Obstructive Lung Disease.

The scatter plot of univariate analysis is shown in Fig. 2. The results of both covariance analysis and the multivariate linear regression were consistent with the univariate analysis presented in Table S4.

### ROCs for FETUB, fibrinogen and their combination

**Figure 3:**
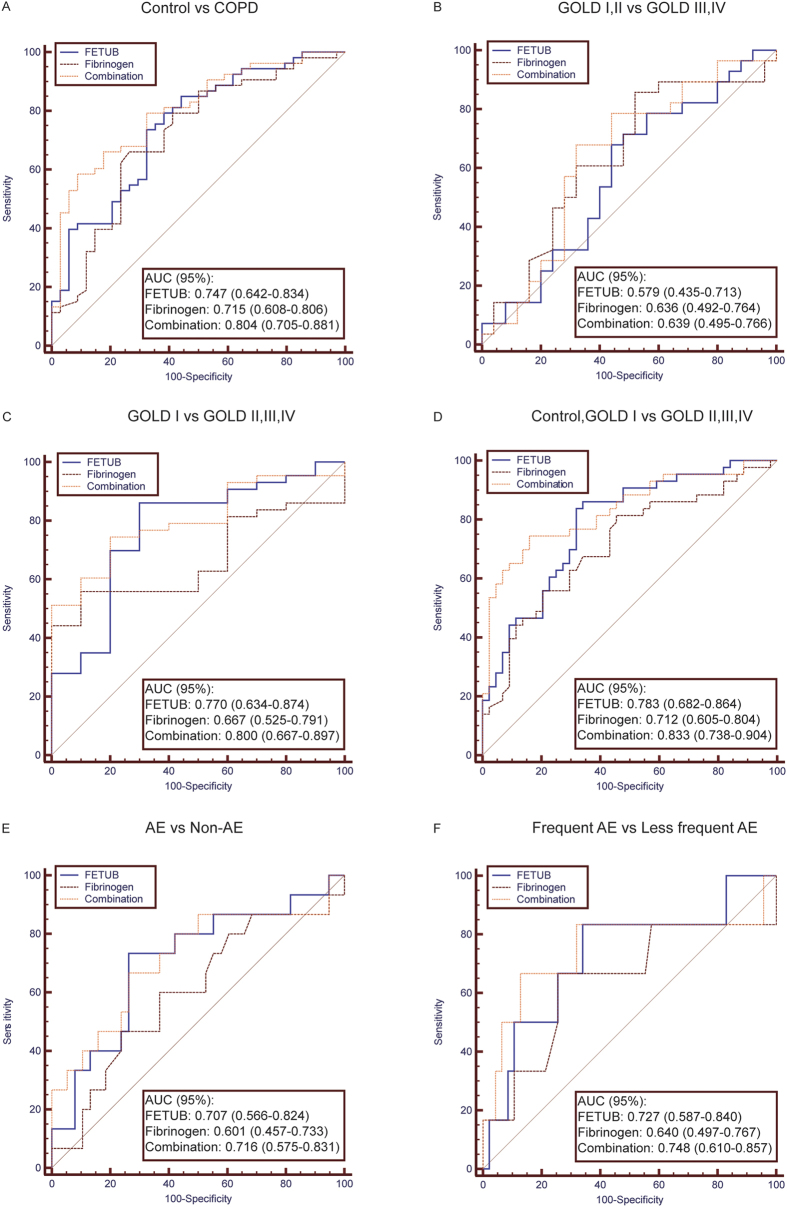
ROCs for FETUB, fibrinogen and their combination in the verification group. Legend: The protein (e.g. FETUB or fibrinogen) with a greater AUC value has the better distinguishing ability for the comparison (e.g. control vs COPD or GOLD I, II vs GOLD III, IV). (**A**) One group includes controls, and the other group includes COPD patients with GOLD I–IV. (**B**) One group includes COPD patients with GOLD I and II, and the other group includes COPD patients with GOLD III and IV. (**C**) One group includes COPD patients with GOLD I, and the other group includes COPD patients with GOLD II, III and IV. (**D**) One group includes controls and COPD patients with GOLD I, and the other group includes COPD patients with GOLD II, III and IV. (**E**) One group includes COPD patients with occurrence of AE, and the other group includes COPD patients without occurrence of AE in a one-year fellow-up. (**F**) One group includes COPD patients with occurrence of frequent AE, and the other group includes COPD patients with occurrence of less frequent AE in a one-year follow-up. Frequent AE: The number of AE of COPD patients per year is greater than two. Less frequent AE: The number of AE of COPD patients per year is 0 or 1. Abbreviations: COPD, chronic obstructive pulmonary disease; GOLD, Global Initiative for Obstructive Lung Disease; FETUB, fetuin-B; ROC, receive operating curve; AUC, area under curve; AE, acute exacerbation.

To assess the diagnostic values in COPD and predictive abilities in the occurrence of AE, 6 different FETUB ROCs were generated in Fig. 3. The ROCs of fibrinogen and their combination were also generated as a reference in each panel. The corresponding areas under the curves (AUCs) and cut-off values are presented in Table S5.

Panel (A) shows that FETUB distinguished COPD from controls (AUC: 0.747, 95% CI: 0.642–0.834) whose AUC was slightly greater than that of fibrinogen (AUC: 0.715, 95% CI: 0.608–0.806). The combination of FETUB and fibrinogen performed better (AUC: 0.804, 95% CI: 0.705–0.881) than FETUB alone.

Panel (B) shows that FETUB, fibrinogen and their combination could not distinguish GOLD I and II from GOLD III and IV for a COPD patient.

Panel (C) shows that FETUB could distinguish GOLD I from GOLD II, III and IV for a COPD patient (AUC: 0.770, 95% CI: 0.634–0.874). Fibrinogen did not distinguish them (AUC: 0.667, 95% CI: 0.525–0.791), and the combination of FETUB and fibrinogen (AUC: 0.800, 95% CI: 0.667–0.897) performed slightly better than FETUB alone.

Panel (D) shows FETUB (0.783, 95% CI: 0.682–0.864) distinguished the controls and GOLD I group from the GOLD II, III and IV group, whose AUC was greater than that of fibrinogen (AUC: 0.712, 95% CI: 0.605–0.804). The combination of FETUB and fibrinogen performed better (AUC: 0.833, 95% CI: 0.738–0.904) than FETUB alone.

Panel (E) shows that baseline FETUB (0.707, 95% CI: 0.566–0.824) predicted the occurrence of AE, but baseline fibrinogen did not (AUC: 0.601, 95% CI: 0.457–0.733). The combination of FETUB and fibrinogen (AUC: 0.716, 95% CI: 0.575–0.831) performed slightly better than FETUB alone.

Panel (F) shows baseline FETUB (0.727, 95% CI: 0.587–0.840) predicted the occurrence of frequent AE, but baseline fibrinogen did not (AUC: 0.640, 95% CI: 0.497–0.767). The combination of FETUB and fibrinogen (AUC: 0.748, 95% CI: 0.610–0.857) performed slightly better than FETUB alone.

### Correlation between FETUB and clinical data (FEV1%pred, RV%pred, RV/TLC%, CT emphysema%, grades of lung function and number of AE)

**Figure 4:**
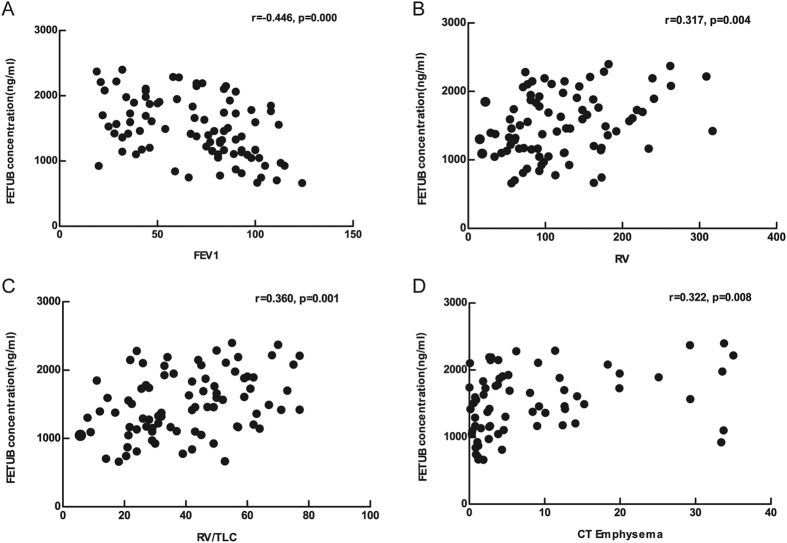
Correlation between FETUB and clinical data in the verification group. Legend: The concentration of FETUB is negatively correlated with (**A**) FEV1 and is positively correlated with (**B**) RV, (**C**) RV/TLC, and (**D**) percentage of emphysema assessed by CT. Abbreviations: FETUB, fetuin-B; FEV1, forced expiratory volume in a second; RV, residual capacity; TLC, total lung capacity; CT, computed tomography.

To assess the correlation between FETUB and variables (FEV1%pred, RV%pred, RV/TLC% and CT emphysema%), Pearson correlation analysis was performed, and the corresponding scatter diagram is shown in Fig. 4. To assess the correlation between FETUB and variables (grades of lung function and number of AE), Spearman correlation analysis was performed. The concentration of FETUB was negatively correlated with FEV1%pred (*r* = −0.446, *p* = 0.000), and positively correlated with RV%pred (*r* = 0.317, *p* = 0.004), RV/TLC% (*r* = 0.360, *p* = 0.004), CT emphysema% (*r* = 0.322, *p* = 0.008), grades of lung function (*r* = 0.456, *p* = 0.000) and number of AE (*r* = 0.326, *p* = 0.017).

The multivariable multivariate linear regression model, which corrected for age, BMI, smoking history, smoking cessation, diabetes mellitus, hypertension, hyperlipidermia, hepatitis, abnormal ALS and AST, demonstrated that FETUB was independently correlated with FEV1%pred, RV%pred, RV/TLC%, CT emphysema% and grades of lung function and the model showed a correlation trend with the number of AE (*p* = 0.072). Details regarding the univariate and multivariate analyses are shown in Table S6.

## Discussion

In the present study, plasma FETUB was identified in COPD by the gel-free iTRAQ-based proteomic technique. Our study found that the FETUB concentrations of COPD patients were significantly greater than that of controls. Our data also indicated FETUB concentrations of the GOLD II, III and IV groups were greater than those the control and GOLD I groups. Howerver, there was not siginificantly different between the GOLD III and GOLD I groups, which may have been due to the lack of statistical power. The concentration of FETUB was not statistically different between the control and GOLD I groups. FETUB was also correlated with important clinical data (FEV1%pred, RV%pred, RV/TLC%, CT emphysema% and grades of lung function). ROCs indicated FETUB had a greater ability to identify more severe grades of lung function in COPD patients and had a greater predictive ability in the occurrence of AE or frequent AE than fibrinogen, and their combination performed better than FETUB or fibrinogen alone.

In our discovery group, differentially expressed proteins in the GOLD II vs GOLD I, GOLD III vs GOLD I and GOLD IV vs GOLD I comparisons were listed in Appendixes I, II and III, respectively. Several of the proteins such as serum amyloid A (SAA)^15^, apolipoprotein^11^ and peroxiredoxin-2^12^ have been previously reported, demonstrating the proteomic approach is reliable for identifying proteins. In addition, the fold changes of both FETUB and GRP-78 determined by gel-free iTRAQ-based proteomic technique were consistent with that determined by ELISA in the discovery group, indicating the quantification ability of the proteomic approach is also reliable.

Recently, a biomarker study^9^ also reported the existence of FETUB in the plasma of COPD patients by a labelling-free untargeted proteomic approach. But they did not show increased FETUB levels in COPD, which disagrees with the present study. However, their conclusion was dependent on the protein expression profile data from 20 subjects and was not verified by ELISA. Generally, a proteomic approach can identify existence of proteins but it requires verification by an immunoassay (e.g. ELISA, Western blot and immunohistochemistry)^19^. For this reason, we investigated the clinical value of proteins by ELISA.

FETUB, a liver-derived plasma protein^20^, has recently been reported to influence glucose metabolism^21^. Thus, a multivariate model, which corrected for ALT, AST, diabetes mellitus and hepatitis history, was used to assess the clinical value of FETUB in COPD.

Our study found the FETUB levels of COPD patients were significantly greater than those of controls and ROCs also indicated that FETUB had the potential to distinguish COPD patients from controls. However, the discernibility was weak since a quite large overlap existed. Of interest, the combination of FETUB and fibrinogen showed better. Thus, FETUB might assist the diagnosis and management of COPD as a complement for other markers.

Our data also showed that the FETUB levels of GOLD II, III and IV were greater than those of both GOLD I and control. ROCs also showed FETUB distinguished GOLD I from GOLD II, III and IV. These results indicate that it can identify the more severe grades of COPD and might monitor the progression of patients with GOLD I. FETUB can not differentiate GOLD I from controls, implying it is not helpful for early diagnosis of COPD.

To investigate the predictive ability of FETUB in the occurrence of AE or frequent AE, we conducted a one-year follow-up study in the verification group. According to results of the ROCs and correlation analyses, baseline FETUB can predict the occurrence of AE or frequent AE and it showed a correlation trend with the number of AE in the following year, indicating its levels have the potential to reflect the progression of disease.

To further explore the potential effect of FETUB in COPD, we conducted correlation analyses between FETUB and some important clinical data (FEV1%pred, RV%pred, RV/TLC%, CT emphysema% and grades of lung function). Our study found that FETUB was positively correlated to FEV1%pred and grades of lung function, indicating it can reflect the severity of lung function or airway limitation. Currently, the biological function of FETUB in diseases is little known. However, fetuin-A, another member of the fetuin family, has been studied widely^22^,^23^ and found to play a significant role in the regulation of inflammatory signalling by activating Toll-like receptors^24^ and is also an endogenous inhibitor of human meprin metalloproteases^25^. Owing to structural similarities^20^, we propose that the biological activity of FETUB may be similar to fetuin-A and may be involved in the inflammatory progression of COPD including airway inflammation. FETUB was also associated with RV%pred, RV/TLC% and CT emphysema%, indicating it can reflect the severity of emphysema. The imbalance of protease-antiprotease plays a major role in the progress of emphysema^26^. A high-quality study^27^ has demonstrated FETUB sustains fertilization by inhibiting the protease activity of ovastacin, a cortical granula protease known to trigger zona pellucida hardening. For this reason, FETUB, a member of the cystatin superfamily, may play a role in the development of emphysema. Herein, it is valuable to explore the potential mechanism of FETUB in airway inflammation or emphysema of COPD.

Our work has the following strengths. First, this is the first study using iTRAQ-based proteomic technique to identify plasma biomarkers of COPD. Second, our study design is different from other proteomic study strategies and it may help find new biomarkers. We first tried to find biomarkers among COPD patients with different grades of lung function by proteomics and then determined its difference between COPD patients and controls by ELISA. Generally, other studies first tried to find biomarkers between COPD and controls by proteomics and then determined its difference among COPD patients with different grades of lung function by immunoassay (e.g. ELISA, western blot and immunohistochemistry). Third, we set strict inclusion criteria to ensure the matching of clinical variables among all groups. For mismatching variables, covariance analyses and multivariate regression model were performed to correct them. Fourth, we also verified the biomarker in another independent population, increasing the reliability of results. Finally, we also performed a one-year follow-up study, broadening its clinical value.

Our study has several limitations. First, the discovery group only consisted of 8 COPD patients because iTRAQ-based proteomic technique only labels 8 different samples in a same pool. To reduce the potential bias, we verified the proteomic results in a larger population by ELISA. Second, the control group was not set in the discovery group due to the limitation of sample size of the proteomic approach. Third, no female subject is included in this study because nearly all COPD patients in China are male. Forth, the new classificaton system is not used in the study because the aim of the study is to seek biomarkers related to the severity of lung function. Finally, its levels were not measured when AE occurred because information of patients was acquired by telephone, and the follow-up period is short, which restricted further analyses (e.g., mortality).

In conclusion, our study found that FETUB could identify more severe grades of lung function in COPD patients and predicted the occurrence of AE or frequent AE. The potential value of FETUB is likely to assist the diagnosis and management of COPD as a complement for other markers.

## Methods

### Study Design and Subject Selection

**Figure 5:**
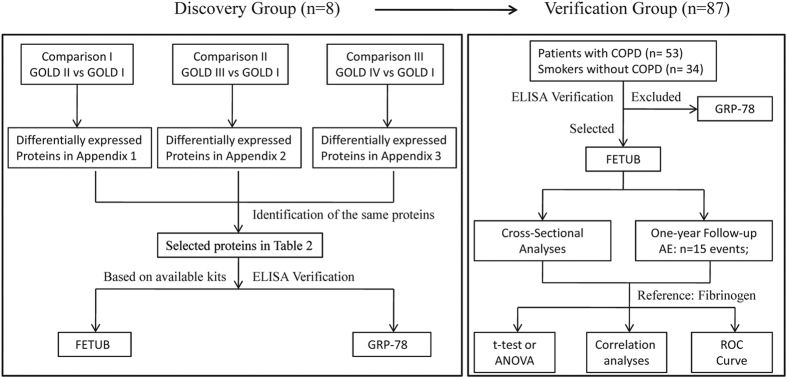
Flow diagram of the study Abbreviations: GOLD, Global Initiative for Obstructive Lung Disease; ELISA, enzyme-linked immuno sorbent assay; FETUB, fetuin-B; GRP-78, 78 kDa glucose-regulated protein; COPD, chronic obstructive pulmonary disease; AE, acute exacerbation; ROC, receiver operating characteristic curve.

Two groups of subjects were enrolled at the Peking University Third Hospital, China. One group is a discovery group including 8 COPD patients (n = 2, GOLD I; n = 2, GOLD II; n = 2, GOLD III; n = 2, GOLD IV). The set of subjects were recruited in January 2013 to identify plasma biomarker candidates by gel-free iTRAQ-based proteomic technique. Another group was a verification group including 34 smokers and 53 COPD patients (n = 10 GOLD I; n = 15 GOLD II; n = 18 GOLD III; n = 10 GOLD IV). The set of subjects were recruited from January to March 2015 to verify the plasma biomarker candidates by ELISA. To collect AE information for these subjects, they were interviewed by telephone every 6 months after inclusion by telephone until February 2016. The mean follow-up period of subjects was one year. The flow diagram of the study is shown in Fig. 5. The study protocol was approved by Peking University Institutional Review Board Office (PUIRB) and the study was strictly conducted according to the protocol. Written informed consent was acquired from all subjects.

The COPD patients in this study were included if they met the following parameters: 1) males aged 50–70; 2) diagnosed with COPD according to the GOLD guidelines^1^; 3) clinically stable patients without medication changes or exacerbation in two months; and 4) smoking history of more than 10 pack years and smoking cessation of more than 5 years. Selected COPD patients were excluded if they met the following parameters: 1) diagnosed with unstable cardiovascular diseases, significant renal or hepatic dysfunction or mental incompetence; 2) diagnosed with asthma, active pulmonary tuberculosis, diffuse panbronchiolitis, cystic fibrosis, clinically significant bronchiectasis, exacerbation of COPD or pneumonia in two months; or 3) prescribed immunosuppressive medications. A matched group of male smokers of similar age, smoking history and smoking cessation without COPD were included as controls. According to FEV1%pre after a bronchodilator, the severity of lung function was classified into four grades as follows: GOLD I (FEV1%pre ≥ 80%), GOLD II (50% ≤ FEV1%pre < 80%), GOLD III (30% ≤ FEV1%pre < 50%) and GOLD IV (FEV1%pre < 80%)^1^.

According to GOLD guidelines, AE is an acute event characterized by a worsening of the patient’s respiratory symptoms that is beyond normal day-to-day variations and leads to a change in medication, and frequent AE is ≥2 exacerbations per year^1^. None of the included subjects were hospitalized due to AE.

The percentage of emphesema was assessed using a chest CT scan with an attenuation value of less than −950 houndsfield units (HU) using AW version 4.5 software (GE healthcare, fairfield, CT, USA)^28^. The index was called “CT emphysema%”.

Age, gender, height, weight, smoking history, smoking cessation, concomitant diseases (diabetes mellitus, hyperlipidermia, hepatitis and hypertension) were collected by inquiring subjects. FEV1%pred, RV%pred, and RV/TLC% were acquired by pulmonary function test (SensorMedics, Yorba Linda, CA, USA).

### Sample Collection

Blood samples (approximately 5 mL) were collected from each subject via an antecubital vein into a vacuum tube containing Ethylene Diamine Tetraacetic Acid (EDTA). Blood sample was centrifuged at room temperature within half an hour. The separated plasma sample was aliquoted into sterile microcentrifuge tubes (500 μl each vial) and then were stored at −80 °C.

### Gel-free iTRAQ-based Proteomic Analysis

The highly abundant proteins in plasma in the discovery group were depleted using ProteoMinerTM kits (Bio-Rad Laboratories, Hercules, CA, USA) according to the manufacturer’s protocol.

The proteins were extracted out of plasma of each subject and were digested with Trypsin Gold (Promega, Madison, WI, USA) at 37 °C for 16 hours. Then peptides were processed according to the manufacture’s protocol for 8-plex iTRAQ reagent (Applied Biosystems, Carlsbad, CA, USA). The peptides of each subject were labeled with different iTRAQ tags respectively and were incubated at room temperature for 2 h. Then the labeled samples were pooled and dried by vacuum centrifugation.

Strong cation exchange chromatography (SCX) was conducted with a LC-20AB HPLC Pump system (Shimadzu, Kyoto, Japan). The peptides were subjected to nanoelectrospray ionization followed by liquid chromatography coupled with tandem mass spectrometry (LC-MS/MS) in an Q EXACTIVE (Thermo Fisher Scientific, San Jose, CA, USA).

Proteins were identified in the Uniprot_human database by Mascot search engine version 2.3.02 (Matrix Science, London, UK). All proteomic and bioinformatic analyses were performed by BGI (Shenzhen, China).

### Immunoassay Analysis

FETUB (abcam^®^, Cambridge, MA, USA) and GRP-78 (BioCell PC, Enzo Life Sciences AG, Switzerland) were measured by ELISA in all subjects using a 96-well plate immunoassay kit according to the manufacturers’ protocol. Plasma fibrinogen concentration was measured by the Clauss method^29^. Concentrations of ALT and AST were measured by a biochemistry automatic analyser (Roche Diagnostics, Branchburg, NJ, USA).

### Statistical Analysis

Normal distribution of continuous variables in each group was assessed by Shapiro-Wilk (S-W) test. Thier variance homogeneity was assessed by Levene test. If *p* values of both S-W test and Levene test were greater than 0.1, ANOVA or t-test would be conducted. Otherwise, Kruskal-Wallis H test or Mann-Whitney U test would be performed. Continuous variables ALT and AST were transformed into the categorical variables according to the cut-off value (40 IU/L). ALT and AST, greater than 40 IU/L, were defined as “abnormal”. Categorical variables were assessed by chi square test.

If the difference of covariables (age, BMI, smoking history and smoking cessation, diabetes mellitus, hyperlipidermia, hepatitis and hypertension) in each group was significant statistically, the covariables were considered to influence the targeted biomarkers (eg. FETUB) as a potential confounder. Then analyses of covariance and multivariate linear regression model, which corrected these confounders, were then conducted and a post-hoc test was used for comparisons between groups.

To determine the relationship between FETUB concentration and important clinical data (FEV1%pred, RV%pred, RV/TLC%, CT emphysema%, grades of lung function and number of AE), Pearson or Spearman correlation analyses with corresponding scatter plot and multivariate linear regression model were conducted. To determine the capability of FETUB in distinguishing COPD from controls and identifying the grades of lung function of COPD patients and to determine its capability in predicting the occurrence of AE or frequent AE, receiver operating characteristic curve (ROC) of FETUB was performed. The area under curve (AUC) and cut-off value with corresponding sensitivity and specificity were calculated. As a reference, ROCs of fibrinogen and ROCs of the combination of FETUB and fibrinogen were also performed with corresponding AUCs. Statistical analyses were conducted using SPSS version 19 software (Statistics Package for the Social Sciences, Chicago, IL, USA), GraphPad Prism version 5.0 software (GraphPad, San Diego, CA, USA) and MedCalc^®^ version 15.10.0 (MedCalc, Ostend, Belgium).

## Additional Information

**How to cite this article**: Diao, W.-q. *et al*. Fetuin-B (FETUB): a Plasma Biomarker Candidate Related to the Severity of Lung Function in COPD. *Sci. Rep*. **6**, 30045; doi: 10.1038/srep30045 (2016).

## Supplementary Material

Supplementary Information

Supplementary Table S1

Supplementary Table S2

Supplementary Table S3

## Figures and Tables

**Table 1:** Characteristics of the verification group.

Variable	COPD vs Control		GOLD I, II, III, IV vs Control			
	Control (n = 34)	COPD (n = 53)	GOLD I (n = 10)	GOLD II (n = 15)	GOLD III (n = 18)	GOLD IV (n = 10)
Age (yrs)	60 (58–63)	64 (61–68)**	65 (63–67)	66 (61–70)	64 (61–67)	64 (60–67)
BMI (kg/m^2^)	24 ± 3	24 ± 3	25 ± 2	25 ± 4	23 ± 3	23 ± 3
Smoking history (pack-yrs)	36 (33–40)	37 (30–46)	37 (32–42)	40 (33–48)	30 (24–46)	39 (29–50)
Smoking cessation (yrs)	10.7 ± 2.2	11.8 ± 3.2	10 (7–13)	14 (13–16)++	10 (9–13)	11 (10–13)
FEV1 (% pred)	95 (86–104)	47 (35–72)**	83 (82–85)	68 (59–70)++	40 (35–45)++	24 (21–29)++
RV (% pred)	77 (58–97)	137 (91–185)**	66 (53–106)	115 (81–154)	163 (124–185)++	262 (209–309)++
RV/TLC (%)	26.5 ± 10.8	49.3 ± 16.3**	33 (29–44)	42 (34–50)++	56 (47–62)++	73 (68–77)++
CT Emphysema† (%)	1.4 (0.77–2.7)	11.4 (4.8–19.9)**	4.8 (2.3–5.2)	5.3 (2.4–9.8)	12.7 (10.1–25.1)++	29.3 (12.6–33.4)++
AE$	None	15 (28.3%)	0 (0%)	5 (33.3%)	6 (33.3%)	4 (40.0%)
Frequent AE$ (≥2 per year)	None	6 (11.3%)	0 (0%)	2 (13.3%)	2 (11.1%)	2 (20.0%)
FETUB (ng/ml)	1257 ± 414	1652 ± 427**	1345 ± 391	1762 ± 427++	1650 ± 375++	1800 ± 451++
Fibrinogen (g/L)	2.5 (2.3–3.0)	3.1 (2.7–3.7)**	3.0 (2.5–3.1)	2.8 (2.5–4.0)	3.2 (2.9–3.6)	3.7 (2.9–4.1)+
GRP-78 (μg/ml)	5.8 ± 1.5	6.0 ± 1.4	5.9 ± 1.3	6.2 ± 1.3	5.8 ± 1.6	6.0 ± 1.5
Diabetes Mellitus	1 (2.9%)	5 (9.4%)	1 (10%)	2 (13.3%)	1 (5.6%)	1 (10%)
Hyperlipidermia	1 (2.9%)	2 (3.8%)	0 (0%)	1 (6.7%)	1 (5.6%)	0 (0%)
Hepatitis	1 (2.9%)	0 (0%)	0 (0%)	0 (0%)	0 (0%)	0 (0%)
Hypertension	2 (5.9%)	16 (30.2%)**	2 (20%)	6 (40%)	6 (33.3%)	2 (20%)
Abnormal ALT^#^	0 (0%)	2 (3.8%)	0 (0%)	1 (6.7%)	1 (5.6%)	0 (0%)
Abnormal AST^#^	3 (8.8%)	3 (5.7%)	0 (0%)	1 (6.7%)	2 (11.1%)	0 (0%)

^*^p < 0.05 or.

^**^p < 0.01: COPD patients were compared with these of controls.

^+^p < 0.05 or

^++^p < 0.01: The levels of of GOLD I, II, III and IV were compared with these of controls respectively.

^†^CT Emphysema: percentage of emphysema assessed by CT.

^$^AE or frequent AE: The information was collected in a one-year fellow-up.

^#^Abnormal ALT: The number of subjects whose ALT was greater than 40 IU/L. ^#^Abnormal AST: The number of subjects whose AST was greater than 40 IU/L. Abbreviations: COPD, chronic obstructive pulmonary disease; GOLD, Global Initiative for Obstructive Lung Disease; BMI, body mass index; FEV1, forced expiratory volume in a second; RV, residual capacity; TLC, total lung capacity; CT, computed tomography; AE, acute exacerbation; FETUB, fetuin-B; GRP-78, 78 kDa glucose-regulated protein; ALT: alanine aminotransferase; AST: aspartate aminotransferase.

**Table 2:** Identification of the same proteins in (GOLD II vs GOLD I), (GOLD III vs GOLD I) and (GOLD IV vs GOLD I).

Accession	Abbreviation	Protein Name	Fold change*		
			GOLD II vs GOLD I	GOLD III vs GOLD I	GOLD IV vs GOLD I
P11021	GRP-78	78 kDa glucose-regulated protein	0.7125	–	0.5255
P07360	CO8G	Complement component C8 gamma chain	0.65	–	0.488
Q14195	DPYL3	Dihydropyrimidinase-related protein 3	0.5225	–	0.3245
P04406	G3P	Glyceraldehyde-3-phosphate dehydrogenase	0.8305	–	0.5925
P01608	KV116	Ig kappa chain V-I region Roy	0.6845	–	0.604
P09972	ALDOC	Fructose-bisphosphate aldolase C	0.653	–	0.383
Q9UGM5	FETUB	Fetuin-B	0.634	1.613	1.4855
P01877	IGHA2	Ig alpha-2 chain heavy chain constant region	1.531	–	2.271
Q8TDL5	BPIB1	BPI fold-containing family B member 1	1.5075	1.7835	–
Q9Y6Z7	COL10	Collectin-10	1.423	–	1.227

The protein is not quantified or the fold change is not significant statistically in this comparison.

^*^Fold change: GOLD II, III or IV is compared to GOLD I, respectively.
