# Supplementary material for: Fetuin-B (FETUB): a Plasma Biomarker Candidate Related to the Severity of Lung Function in COPD
Source: Sci Rep. 2016 Jul 22;6:30045. doi: 10.1038/srep30045 (PMC4957096; doi:10.1038/srep30045)
Supplement: Supplementary Information [file srep30045-s1.doc]

**Title Pages**

1. **The title of the paper**:

Fetuin-B (FETUB): a Plasma Biomarker Candidate Related to the Severity of Lung Function in COPD in COPD

1. **The full names of the authors:**

Wen-qi Diao^1^, Ning Shen^1^, Yi-peng Du^1^, Bei-bei Liu^1^, Xiao-yan Sun^1^, Ming Xu^2^, Bei He^1*^.

1. **The  affiliations of the authors:**

3-1. Department of Respiratory Medicine, Peking University Third Hospital, Beijing 100191, China.

3-2. Department of Cardiology, Institute of Vascular Medicine, Peking University Third Hospital, Key Laboratory of Molecular Cardiovascular Sciences, Ministry of Education, Beijing 100191, China.

1. **E-mail address of the authors**

Wen-qi Diao^1^, 424259600@qq.com; Ning Shen^1^, [shenning1972@126.com](mailto:shenning1972@126.com); Yi-peng Du^1^, [billydu@sina.com](mailto:billydu@sina.com); Bei-bei Liu^1^, [liubeibei416@126.com](mailto:liubeibei416@126.com); Xiao-yan Sun^1^, [923474143@qq.com](mailto:923474143@qq.com); Ming Xu^2^, [xuminghi@bjmu.edu.cn](mailto:xuminghi@bjmu.edu.cn); Bei He^1*^, puh3_hb@bjmu.edu.cn.

1. *** Corresponding author**

Bei He, M.D./Prof.

E-mail: [puh3_hb@bjmu.edu.cn](mailto:puh3_hb@bjmu.edu.cn)

Address: Department of Respiratory Medicine, Peking University Third Hospital, Beijing, 100191, China.

Fax：86 01082086810
Telephone：86 01082265210

Table S1: Characteristics of the discovery group

| Variable | GOLD I (n=2) | GOLD II (n=2) | GOLD III (n=2) | GOLD IV (n=2) |
| --- | --- | --- | --- | --- |
| Age (yrs) | 56±6 | 59±2 | 57±8 | 62±4 |
| BMI (kg/m^2^) | 25±0 | 25±3 | 25±0 | 23±0 |
| Smoking history (pack-yrs) | 39±2 | 39±4 | 39±2 | 37±0 |
| Smoking cessation (yrs) | 11±3 | 12±0 | 14±2 | 14±1 |
| FEV1 (% pred) | 81±1 | 70±11 | 43±2 | 21±8 |
| RV (% pred) | 82±14 | 94±8 | 116±16 | 286±33 |
| RV/TLC (% pred) | 30±4 | 38±6 | 47±1 | 69±1 |
| CT Emphysema^†^ (%) | 5.0±0.30 | 4.5±2.4 | 11.3±1.6 | 23.0±14.7 |
| FETUB (ng/ml) | 1255±70 | 976±190 | 1480±358 | 1572±322 |
| GRP-78 (μg/ml) | 6.5±0.78 | 5.3±0.45 | 6.2±0.34 | 4.7±0.91 |

† CT Emphysema: percentage of emphysema assessed by CT

Abbreviations: COPD, chronic obstructive pulmonary disease; GOLD, Global Initiative for Obstructive Lung Disease; BMI, body mass index; FEV1, forced expiratory volume in a second; RV, residual capacity; TLC, total lung capacity; CT, computed tomography; FETUB, fetuin-B; GRP-78, 78 kDa glucose-regulated protein;

| Variable | COPD vs Control | | | | |  | GOLD I, II, III, IV and Control | | | | | | | |
| --- | --- | --- | --- | --- | --- | --- | --- | --- | --- | --- | --- | --- | --- | --- |
|  | S-W Test | |  | Levene  test | Comparison Method |  | S-W Test | | | | |  | Levene  Test | Comparison Method |
|  | Control  n=34 | COPD  n=53 |  |  |  |  | Control  n=34 | GOLD I  n=10 | OLD II  n=15 | GOLD III  n=18 | GOLD IV  n=10 |  |  |  |
| Age | *p*=0.27 | *p*=0.07 |  | *p*=0.49 | *p*=0.00, Mann-Whitney U |  | *p*=0.27 | *p*=0.88 | *p*=0.77 | *p*=0.68 | *p*=0.02 |  | *p*=0.16 | *p*=0.00, Kruskal-Wallis H |
| BMI | *p*=0.81 | *p*=0.17 |  | *p*=0.62 | *p*=0.91, T-Test |  | *p*=0.81 | *p*=0.46 | *p*=0.25 | *p*=0.81 | *p*=0.47 |  | *p*=0.60 | *p*=0.39, ANOVA |
| Smoking history | *p*=0.76 | *p*=0.00 |  | *p*=0.01 | *p*=0.64, Mann-Whitney U |  | *p*=0.76 | *p*=0.84 | *p*=0.482 | *p*=0.00 | *p*=0.30 |  | *p*=0.00 | *p*=0.31, Kruskal-Wallis H |
| Smoking cessation | *p*=0.40 | *p*=0.24 |  | *p*=0.04 | *p*=0.07, Adjusted T-Test |  | *p*=0.40 | *p*=0.23 | *p*=0.09 | *p*=0.08 | *p*=0.43 |  | *p*=0.26 | *p*=0.00, Kruskal-Wallis H |
| FEV1 | *p*=0.99 | *p*=0.06 |  | *p*=0.00 | *p*=0.00, Mann-Whitney U |  | *p*=0.99 | *p*=0.03 | *p*=0.69 | *p*=0.24 | *p*=0.02 |  | *p*=0.00 | *p*=0.00, Kruskal-Wallis H |
| RV | *p*=0.04 | *p*=0.49 |  | *p*=0.01 | *p*=0.00, Mann-Whitney U |  | *p*=0.04 | *p*=0.03 | *p*=0.92 | *p*=0.99 | *p*=0.76 |  | *p*=0.53 | *p*=0.00, Kruskal-Wallis H |
| RV/TLC | *p*=0.15 | *p*=0.42 |  | *p*=0.01 | *p*=0.00, Adjusted T-Test |  | *p*=0.15 | *p*=0.60 | *p*=0.73 | *p*=0.48 | *p*=0.03 |  | *p*=0.34 | *p*=0.00, Kruskal-Wallis H |
| CT Emphysema^†^ | *p*=0.03 | *p*=0.00 |  | *p*=0.00 | *p*=0.00, Mann-Whitney U |  | *p*=0.03 | *p*=0.17 | *p*=0.14 | *p*=0.02 | *p*=0.31 |  | *p*=0.00 | *p*=0.00, Kruskal-Wallis H |
| FETUB | *p*=0.13 | *p*=0.19 |  | *p*=0.63 | *p*=0.00, T-Test |  | *p*=0.13 | *p*=0.34 | *p*=0.20 | *p*=0.66 | *p*=0.58 |  | *p*=0.86 | *p*=0.00, ANOVA |
| Fibrinogen | *p*=0.06 | *p*=0.01 |  | *p*=0.30 | *p*=0.00, Mann-Whitney U |  | *p*=0.06 | *p*=0.25 | *p*=0.11 | *p*=0.11 | *p*=0.43 |  | *p*=0.11 | *p*=0.01, Kruskal-Wallis H |
| GRP-78 | *p*=0.25 | *p*=0.14 |  | *p*=0.57 | *p*=0.50, T-Test |  | *p*=0.25 | *p*=0.43 | *p*=0.71 | *p*=0.16 | *p*=0.66 |  | *p*=0.82 | *p*=0.88, ANOVA |

Table S2: The distribution, [homogeneity](javascript:void(0);) [of](javascript:void(0);) [variance](javascript:void(0);) test and comparison method between groups of continuous variables in the verification group

† CT Emphysema: percentage of emphysema assessed by CT

Abbreviations: COPD, chronic obstructive pulmonary disease; GOLD, Global Initiative for Obstructive Lung Disease; BMI, body mass index; FEV1, forced expiratory volume in a second; RV, residual capacity; TLC, total lung capacity; CT, computed tomography; FETUB, fetuin-B; GRP-78, 78 kDa glucose-regulated protein;

Table S3: The comparison method of categorical variables between groups in the verification group

|  | COPD vs Control |  | GOLD I, II, III, IV and Control |
| --- | --- | --- | --- |
| Diabetes Mellitus | *p*=0.46, continuity corrected Chi square test |  | *p*=0.50, Fisher’s exact test |
| Hyperlipidermia | *p*=1.00, continuity corrected Chi square test |  | *p*=0.91, Fisher’s exact test |
| Hepatitis | *p*=0.38, Fisher’s exact test |  | *p*=1.00, Fisher’s exact test |
| Hypertension | *p*=0.01, Pearson Chi square test |  | *p*=0.02, Fisher’s exact test |
| Abnormal ALT^#^ | *p*=0.52, Fisher’s exact test |  | *p*=0.37, Fisher’s exact test |
| Abnormal AST^#^ | *p*=0.89, continuity corrected Chi square test |  | *p*=0.91, Fisher’s exact test |

#Abnormal ALT: The number of subjects whose ALT was greater than 40 IU/L

#Abnormal AST: The number of subjects whose AST was greater than 40 IU/L

Abbreviations: ALT: alanine aminotransferase; AST: aspartate aminotransferase

Table S4: The difference of FETUB levels between groups by covariance analysis and multivariate linear regression model

|  | Covariance Analysis | Multivariate linear regression |
| --- | --- | --- |
| **Comparison I (COPD vs Control)** |  |  |
| Group (COPD vs Control) | *p*=0.00 | *p*=0.00 |
| Age | *p*=0.33 | *p*=0.33 |
| Hypertension | *p*=0.35 | *p*=0.35 |
| **Comparison II (GOLD I, II, III, IV, Control)** |  |  |
| Group (GOLD I, II, III, IV vs Control) | *p*=0.00 | *p*=0.00 |
| Smoking cessation | *p*=0.70 | *p*=0.18 |
| Age | *p*=0.31 | *p*=0.43 |
| Hypertension | *p*=0.42 | *p*=0.28 |

Covariance analysis and multivariate linear regression model, correcting age, hypertension and/or smoking history between groups, indicated the group (COPD vs Control or GOLD I, II, III, IV vs Control) is the unique variable influencing FETUB levels.

Abbreviations: COPD, chronic obstructive pulmonary disease; GOLD, Global Initiative for Obstructive Lung Disease

Table S5: Area under curve and cut-off value of FETUB, fibrinogen and their combination

|  | Area Under Curve (95% Confidence Interval) | | |  | Cut-off Value (Sensitivity, Specifity) | |
| --- | --- | --- | --- | --- | --- | --- |
|  | FETUB | Fibrinogen | Combination |  | FETUB | Fibrinogen |
| COPD vs Control | 0.747 (0.642-0.834) | 0.715 (0.608-0.806) | 0.804 ( 0.705-0.881) |  | 1375 ng/ml (73.6%, 67.6%) | 2.9 g/L (66.0%, 73.5%) |
| GOLD I,II vs GOLD III,IV | 0.579 (0.435-0.713) | 0.636 (0.492-0.764) | 0.639 (0.495-0.766) |  | 1490 ng/ml (67.9%, 56.0%) | 2.8 g/L (85.7%, 48.0%) |
| GOLD I vs GOLD II,III,IV | 0.770 (0.634-0.874) | 0.667 (0.525-0.791) | 0.800 (0.667-0.897) |  | 1321 ng/ml (86.0%, 70.0%) | 3.1 g/L (55.8%, 90.0%) |
| Control,GOLD I vs GOLD II,III,IV | 0.783 (0.682-0.864) | 0.712 (0.605-0.804) | 0.833 (0.738-0.904) |  | 1329 ng/ml (86.0%, 65.9%) | 2.7 g/L (81.4%, 54.5%) |
| AE vs Non-AE | 0.707 (0.566-0.824) | 0.601 (0.457-0.733) | 0.716 (0.575-0.831) |  | 1833 ng/ml (73.3%, 73.7%) | 3.2 g/L (60%, 63.2%) |
| Frequent AE vs Less frequent AE | 0.727 (0.587-0.840) | 0.640 (0.497-0.767) | 0.748 (0.610-0.857) |  | 1833 ng/ml (83.3%, 66.0%) | 3.6 g/L (66.7%, 74.5%) |

Abbreviations: COPD, chronic obstructive pulmonary disease; GOLD, Global Initiative for Obstructive Lung Disease; FETUB, fetuin-B; AE: acute exacerbation.

Table S6 Relationship between FETUB and clinical data assessed by univariate correlation analysis and multivariate linear regression model

| Variable | Univariate Correlation Analysis | |  | Multivariate Linear Regression Model (Y= FETUB levels) | | | | | | | | | | | | | | | | |
| --- | --- | --- | --- | --- | --- | --- | --- | --- | --- | --- | --- | --- | --- | --- | --- | --- | --- | --- | --- | --- |
|  |  |  |  | FEV1%pred | |  | RV%pred | |  | RV/TLC% | |  | CT Emphysema% | |  | Grades of lung function | |  | Number of AE | |
|  | Pearson correlation | Spearman correlation |  | Beta | P value |  | Beta | P value |  | Beta | P value |  | Beta | P value |  | Beta | P value |  | Beta | P value |
| FEV1%pred | r=-0.446, p=0.000 | - |  | -0.441 | 0.00 |  | - | - |  | - | - |  | - | - |  |  |  |  |  |  |
| RV%pred | r=0.317, p=0.004 | - |  | - | - |  | 0.315 | 0.011 |  | - | - |  | - | - |  |  |  |  |  |  |
| RV/TLC% | r=0.360, p=0.001 | - |  | - | - |  | - | - |  | 0.400 | 0.001 |  | - | - |  |  |  |  |  |  |
| CT Emphysema%^†^ | r=0.322, p=0.008 | - |  | - | - |  | - | - |  | - | - |  | 0.309 | 0.024 |  |  |  |  |  |  |
| Grades of lung function |  | r=0.456, p=0.000 |  |  |  |  |  |  |  |  |  |  |  |  |  | 0.437 | 0.000 |  |  |  |
| Number of AE |  | r=0.326, p=0.017 |  |  |  |  |  |  |  |  |  |  |  |  |  |  |  |  | 0.297 | 0.072 |
| Age | r=0.094, p=0.388 | - |  | -0.112 | 0.345 |  | 0.019 | 0.883 |  | -0.095 | 0.453 |  | -0.069 | 0.633 |  | -0.093 | 0.431 |  | -0.181 | 0.251 |
| BMI | r=-0.019, p=0.859 | - |  | -0.035 | 0.748 |  | -0.065 | 0.580 |  | -0.066 | 0.558 |  | -0.122 | 0.385 |  | -0.026 | 0.811 |  | -0.028 | 0.867 |
| Smoking history | r=0.100, p=0.355 | - |  | 0.022 | 0.834 |  | -0.022 | 0.848 |  | -0.011 | 0.920 |  | -0.016 | 0.899 |  | 0.013 | 0.900 |  | 0.099 | 0.516 |
| Smoking cessation | r=0.183, p=0.095 | - |  | 0.15 | 0.145 |  | 0.145 | 0.198 |  | 0.175 | 0.110 |  | 0.224 | 0.077 |  | 0.132 | 0.205 |  | 0.134 | 0.372 |
| Diabetes Mellitus | - | r=0.092, p=0.396 |  | 0.063 | 0.545 |  | 0.054 | 0.646 |  | 0.057 | 0.616 |  | 0.054 | 0.678 |  | 0.025 | 0.814 |  | 0.086 | 0.606 |
| Hypertension | - | r=0.198, p=0.066 |  | 0.128 | 0.270 |  | 0.137 | 0.286 |  | 0.116 | 0.352 |  | 0.261 | 0.065 |  | 0.123 | 0.293 |  | 0.043 | 0.806 |
| Hyperlipidermia | - | r=-0.088, p=0.419 |  | -0.083 | 0.468 |  | -0.141 | 0.261 |  | -0.147 | 0.224 |  | -0.172 | 0.213 |  | -0.111 | 0.332 |  | -0.171 | 0.324 |
| Hepatitis | - | r=-0.180, p=0.095 |  | -0.155 | 0.133 |  | -0.213 | 0.060 |  | -0.251 | 0.025 |  | -0.195 | 0.117 |  | -0.158 | 0.127 |  | -^*^ | -^*^ |
| Abnormal ALT^#^ | - | r=0.067, p=0.536 |  | 0.061 | 0.658 |  | 0.012 | 0.940 |  | 0.047 | 0.749 |  | 0.091 | 0.586 |  | 0.042 | 0.765 |  | 0.240 | 0.419 |
| Abnormal AST^#^ | - | r=0.079, p=0.464 |  | -0.014 | 0.917 |  | 0.014 | 0.922 |  | -0.020 | 0.890 |  | -0.029 | 0.859 |  | 0.002 | 0.988 |  | -0.144 | 0.627 |

^†^CT Emphysema: percentage of emphysema assessed by CT

#Abnormal ALT: The number of subjects whose ALT was greater than 40 IU/L

#Abnormal AST: The number of subjects whose AST was greater than 40 IU/L

* Hepatitis: For the model with dependent variable FETUB and independent variable number of AE, the variable hepatitis is constant or has missing correlation and thus is deleted.

Abbreviations: FETUB, fetuin-B; FEV1, forced expiratory volume in a second; RV, residual capacity; TLC, total lung capacity; CT, computed tomography; AE, acute exacerbation; BMI, body mass index; ALT: alanine aminotransferase; AST: aspartate aminotransferase
